# Patients' knowledge and perception of anesthesia and the anesthetists: Cross-sectional study

**DOI:** 10.1016/j.amsu.2022.103740

**Published:** 2022-05-11

**Authors:** Nurhusen Riskey Arefayne, Amare Belete Getahun, Tadesse Belayneh Melkie, Nigussie Simeneh Endalew, Yonas Adissu Nigatu

**Affiliations:** Department of Anesthesia, College of Medicine & Health Sciences, University of Gondar, Gondar, Ethiopia

**Keywords:** Knowledge, Perception, Anesthetists, Perioperative care, AOR, Adjusted odds ratio, CI, Confidence interval, COR, Crude odds ratio, ER, Emergency room, ICU, Intensive care unit

## Abstract

**Background:**

Anesthesiology was misconceived as a behind the screen specialty. Despite significant advancements in its scope, anesthesia remains have a poor public image, clients are not even aware of an anesthetist as a medically qualified health professional, particularly in underdeveloped countries. There has been very little study done on the public's awareness of anesthesia in Ethiopia. This study was conducted to assess the level of patients' knowledge and perception of anesthesia and the anesthetists.

**Methods:**

Institution-based, cross-sectional study was conducted from January to May 2021, at the University of Gondar comprehensive specialized hospital, Ethiopia. Consenting participants were asked to answer a standard questionnaire in a personal interview to assess their knowledge about anesthesia and the anesthetist. Descriptive statistic was used to explain the study participants with study variables and to describe their perception about anesthesia and anesthetists. Binary logistic regression analysis was performed to identify the association between the level of knowledge and independent variables. The strength of the association between the independent variables and the level of knowledge was presented by an adjusted odds ratio and 95% Confidence Interval.

**Results:**

A total of 307 study participants were included in this study with a response rate of 100%. Of these 113 (36.8) were males and 194 (63.2) were females. Two hundred twenty (71.7%) study participants answered less than half of the questions correctly and had a poor level of knowledge the rest 87 (28.3%) had a good level of knowledge and the mean knowledge score was1.72 ± 0.45. Previous anesthesia exposure (p value = 0.001) and occupation (p value = 0.022) of participants had statistically significant association with the level of knowledge.

**Conclusion:**

and recommendation: We have found that patients had very limited (poor) knowledge and perception regarding anesthesia and the role of anesthetists. Anesthetists should do a complete pre-anesthetic assessment which helps them to achieve good patient recognition.

## Highlights

• Around two third of the participant had poor knowledge about anesthesia and the anesthetist.
• Previous anesthesia exposure and occupation are among the factors that affect knowledge of the participant.
• Doing complete pre-anesthetic assessment will helps the anesthetist to achieve good patient recognition.

## 1 Introduction

Studies have been conducted throughout the world to assess the public perception of anesthesia and anesthetists but, knowledge and perception of patients and the public regarding the profession remain poor which most of the time-bound to nonexisting fears and concerns related to the rare risks and complications of anesthesia. The reasons suggested for this may be that anesthetists have less contact with a conscious patient than other medical professionals. Even other healthcare personnel and academic clinical staff do not know the role of the anesthesiologist in or outside the operating room in the hospital. The role of the anesthetist and anesthesia has been an undervalued subject, being considered as a ‘behind the screen’ specialty, in which the leading actor is the surgeon and the anesthetist has only a supportive role [[1], [2], [3], [4], [5]].

In a cross-sectional study done in Ghana, less than two-thirds of patients had heard of anesthesia in which most of them had got the information from health education talks they receive at health facilities. Only about a fifth had heard about anesthesia outside a health facility. This implies that there is poor public awareness about anesthesia, which could negatively impact the knowledge and perception of the general public towards the discipline (6).

In different surveys conducted around the world, it has been shown that two out of three patients know that anesthetists are competent health care professionals that perform their duties independently, this also has a meaning that patients in developing countries are having poorer knowledge compared with those in developed countries for reasons like low education level, scarcity of information through the media and the internet [[6], [7], [8], [9]].

A study done by Bhatarai B et al. has shown that even among medical doctors anesthesia has not been shown as an attractive and demanding specialty that most physicians don't want to specialize in the field (10).

So, the image and status of anesthetists both in the eyes of the medical and lay communities has always been a problem. Even though anesthesiology is growing very fast with impressive technological advancements in the last thirty years, the network media doesn't emphasize the role of the anesthetic team for its great contribution to the successful outcome of the surgery. Rather it focuses on rare medico-legal issues surrounding the patent's perioperative complications and bad events. It is well known that there is tremendous health care awareness, especially in developed countries but, there still is limited knowledge about the structure of the medical services and practices especially relating to the operating room services, moreover relating to anesthetic management and the role played by anesthetists. Another group of people that don't know the current depth of anesthetic practice and the teaching potential of the anesthetist are the paramedical and academic staff [[10], [11], [12], [13]].

Beyond the perioperative care of surgical patients, nowadays the scope of anesthesia is extended to emergency room, trauma units, pain management, resuscitation of critically ill patients, and labor pain management. Some of the misconceptions concerning anesthetic practice are that anesthetists are doctors or trained health care professionals and that the anesthetic team is working under the surgical team. This Nigerian study reported that the perception was found to be poorer in males, older patients, and patients in the lower socio-economic groups. But, fortunately, it was reported that the attitude of medical students towards anesthesia is changed with an increase in the desire to specialize in anesthesia after their perception of anesthesia was altered during their anesthesia rotation [11].

Public awareness programs are being arranged and May 25 is the National Anesthesia Day in many developed countries. This day was celebrated to inform the public about the role and training of anesthetists. In India, people have begun to have awareness since the introduction of the consumer protection act which is an act of the parliament of India that came into action in 1986 to protect the interests of consumers. But, still, the general public has little knowledge of the field [12].

Misunderstanding of anesthesia is being expressed in patients with different responses. In a study done in Canada it is found that approximately 20% of respondents were very concerned about brain damage, waking up intraoperatively and memory loss, 12% were concerned about intraoperative death, 9% were worried about postoperative pain, and 12% reported that they are concerned about nausea and vomiting. But, the general public had a good understanding in considering the pre-anesthetic assessment as an important part of preoperative preparation. Brain damage, death, and awareness under general anesthesia remain prevalent, suggesting that preoperative education of patients should be adequate in addressing such concerns and frustrations. Realistic fears like nausea, vomiting, and postoperative discomfort were less prevalent [14].

## 2 Methodology

### 2.1 Study design, period and area

An institution-based, cross-sectional study was conducted from February to May 2021.

The study was conducted in the University of Gondar Hospital surgical, maternity, orthopedic, gynecology, and fistula wards. The University of Gondar Hospital is a comprehensive specialized and teaching hospital which is located in Gondar town. Gondar is the capital of the North-Gondar administrative zone in Amhara regional state, which is located 748 km northwest of the capital Addis Ababa and 230 km from the Ethio-Sudan border. According to the report of the Central Statistical Agency done in 2007, the population of the North Gondar zone is 2,929,628, of this about 333,432 people live in Gondar town [15]. It is one of the largest hospitals in the country which provides health services for more than five million people in the catchment area. The hospital has 500 hundred beds, seven operation theatres, and one medical intensive care unit. According to the annual report of the anesthesia department, 5695 patients were operated upon under anesthesia in 2013 [16]. The article has been registered with UIN 7699 in research registry. This work has been reported in line with the STROCSS criteria [17].

### 2.2 Source population

All patients who came to the University of Gondar Comprehensive Specialized Hospital.

### 2.3 Study population

All patients who are admitted to surgical, orthopedic, maternity, gynecology, and fistula wards.

### 2.4 Inclusion criteria

All patients over 18 years old, admitted to the surgical, orthopedic, maternity, gynecology, and fistula wards, and patients who consented to participate in the study in the University of Gondar Hospital were included in the study.

### 2.5 Exclusion criteria

Patients who are unwilling to participate in the study, patients scheduled for emergency surgery, patients under the age of 18 years patients, with psychiatric problems, patients who are unable to speak, or patients with hearing difficulty were excluded from the study.

## 3 Variables of the study

### 3.1 Dependent variables

Patients' level of knowledge and perception of anesthesia and the role of anesthetists were the dependent variables.

### 3.2 Independent variables

Age, sex, level of education, previous anesthetic/surgical experience, residence, and occupation were the independent variables.

### 3.3 Operational definitions

**Anesthetist:** any qualified health professional who helps patients both inside and outside the opration room in a hospital by applying the principles and practices of anesthesiology. This will include the terms anesthetist and anesthesiologist.

**Anesthesia:** loss of sensation, especially of pain, and memory which is induced by drugs: this is called general anesthesia when consciousness is lost and it is called local/regional anesthesia when only a specific area/region of the body is involved.

**Surgery:** All major operations requiring general or regional anesthesia.

**Knowledge:** the facts known by the participants (patients) gained by experience or learning and participants are considered to have a good knowledge if they correctly answer half or more of the questions regarding anesthesia and anesthetists.

**Perceptions:** the way the participants (patients) think or understand anesthesia or anesthetists.

**Preoperative:** A time to evaluate the patient by the anesthetist before the operation to give and get the necessary information to and from the patient.

## 4 Sample size determination and sampling techniques

### 4.1 Sample size determination

For sample size determination we had used a single population proportion formula.

In a study done in Nigeria, they have used the same formula by using 76% as a proportion and assuming 95% confidence interval and 5% margin of error, we have calculated the sample size as:

n=(Zα2)2ρ(1−ρ)ε2

$$\begin{array}{l} n=\frac{{\left(1.96\right)}^{2}\;\times 0.76\left(1-0.76\right)}{{\left(0.05\right)}^{2}}\\ n=279 \end{array}$$

With a non-response rate of 10%, we finally calculate the sample size to be **307**.

### 4.2 Sampling technique

All consecutive patients who were admitted in surgical, orthopedic, gynecology, maternity, and fistula wards in University of Gondar Comprehensive Specialized Hospital and were also eligible for the study based on the inclusion-exclusion criteria and until the target sample size was achieved have been requested for voluntary participation by signing informed consent.

### 4.3 Data collection procedure

Data was collected using interviewer-administered pre-tested questionnaires. Its face and content validity was checked among seven experts (in the field of anesthesia) and the scale level content validity index by universal agreement was 0.82, scale level content validity index by average was 0.96; which shows excellent content validity. The internal validity of the instrument was also checked (Cronbach alfa = 0.85) which ensures the internal consistency of the instrument. Two data collectors collected the data and one experienced person was supervising the data collection process. Both Amharic and English versions of the questionnaire were prepared and participants were interviewed using the Amharic version by the data collector.

### 4.4 Data quality management

Data collectors and supervisors have got trained by the principal investigator. To ensure the quality of data, a pilot study for pre-testing of the data collection tool (the questionnaire) was conducted on 10 patients who were not included in the main study. Then necessary corrections have been made according to the feedback obtained and the final questionnaire was ready for the main study. The data collectors and supervisors were closely supervised by the principal investigator throughout the study period. Study participants were provided with adequate information regarding assessment tools (the questionnaire). The collected data were checked for completeness, accuracy, and clarity.

### 4.5 Data processing and analysis

First, the data was entered into Epi-Info version 7. Then, data was transferred to and checked in SPSS version 20. Descriptive statistic was used to explain the study participants with study variables. Bivariate analyses have been performed to determine each of the independent variables. Only variables with a p-value less than 0.25 during bivariate analysis were entered into the multivariate analysis. Binary logistic regression analysis was conducted to identify the factors that affect patients' knowledge and perception about anesthesia and the role of anesthetists. The strength of the association between the independent variables and the level of knowledge and perception of patients on anesthesia and the role of anesthetists was presented by odds ratio and 95% Confidence interval. Finally, data were presented by using numbers, frequencies, tables, charts, and figures. A P-value less than 0.05 was considered statistically significant.

### 4.6 Ethical considerations

Ethical clearance to conduct the research was obtained from the Ethical review committee of the college of medicine and health sciences, University of Gondar. Written informed consent form using mother tongue language was presented and consent was taken from each study participant after a brief explanation and full disclosure of the benefit and risk they will get from participation. Every participant is allowed to discontinue participation at any stage of the data collection time. And they were assured that their treatment and other benefits they can gain from the hospital will not be interrupted or reduced due to their withdrawal.

## 5 Result

### 5.1 Socio-demographic characteristics of study participants

A total of 307 study participants were involved in this study with a response rate of 100%. Of these 113 (36.8) were males and 194 (63.2) were females. About 178 (58%), were in the age category between 25 and 54 years, 76 (24.8) between 18 and 24 years, 33 (10.7%) between 55 and 64 and the rest 20 (6.5%) participants were 65 and above years old. Regarding educational status 110 (35.8) were illiterate, 33 (10.7%) can read and write, 39 (12.7%) completed elementary school, about 52 (16.9%) joined high school, 44 (14.3%) were college graduates and the rest 29 (9.4%) were graduated from University. 164 (53.4%) were urban residents and 143 (46.6%) live in rural areas. Most participants make their daily living by farming (116, 37.8%), by doing a private business (62, 20.2%), and government employees were 60 (19.5) and the rest 69 (22.5%) were in the others category which involves housewives, students and even participants with no job.

### 5.2 Previous history of anesthetic exposure

Regarding previous anesthesia exposure, 134 patients responded that they have received anesthetic drugs for different surgical interventions. Among these ninety-eight (73.1%) took anesthesia once, twenty-five (18.6%) took it twice and the rest eleven (8.2%) took it three or more times. General anesthesia was the type of anesthesia received by most patients (62, 46.3%), about 52 (38.8%) patients were exposed to regional anesthesia, 12 (8.95%) took local anesthesia and about 8 (5.97%) patients did not remember or don't know the type of anesthetic they have taken (Table 1).

**Table 1:** Socio-demographic profiles and previous anesthesia history.

Variables	Frequency	Percent (%)
Age (grouped in years)		
18-24	76	24.8
25-54	178	58
55-64	33	10.7
≥ 65	20	6.5
Sex		
Male	113	36.8
Female	194	63.2
Educational status		
None (illiterate)	110	35.8
Can read and write	33	10.7
Elementary school	39	12.7
High school	52	16.9
College	44	14.3
University	29	9.4
Residence		
Urban	164	53.4
Rural	143	46.6
Occupation		
Government employee	60	19.5
Private business	62	20.2
Farming	116	37.8
Others	69	22.5
Previous anesthesia exposure		
Exposed	134	43.6
Non exposed	173	56.4
Frequency of exposure		
Once	98	73.1
Two times	25	18.6
Three or more times	11	8.2
Type of anesthetic received		
General anesthesia	62	46.3
Regional anesthesia	52	38.8
Local anesthesia	12	8.95
I don't remember (I don't know)	8	5.97

### 5.3 Level of knowledge of participants about anesthesia and the anesthetists

**Fig. 1:**
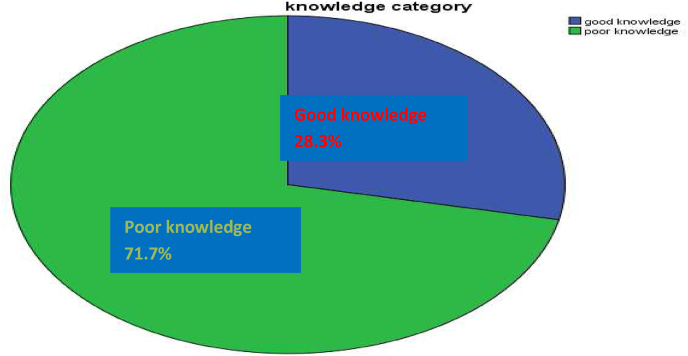
Level of knowledge of participants.

Out of the 22 knowledge measuring questions about 220 (71.7%), study participants answered only 11 and fewer questions correctly and had a poor level of knowledge about anesthesia and the role of anesthetists and only the rest 87 (28.3%) had a good level of knowledge who had answered 12 or more questions correctly and the mean knowledge score was1.72 ± 0.45 (Fig. 1).

In a binary logistic regression variables with p value less than or equal to 0.25 such as educational status (p value = 0.005), residence (p value = 0.031), occupation status of participants (p value = 0.001), previous anesthetic history (p value < 0.001) and frequency of anesthesia exposure (p value = 0.001) were entered into a multi-variate analysis and only variables like previous anesthesia exposure (p value = 0.001) and occupation (p value = 0.022) of participants had effect on the level of knowledge with a statistically significant results.

There were about 194 female participants and among these 138 had a poor level of knowledge and 56 had good knowledge about anesthesia and anesthetists. Male participants were about 113 and of these 82 had poor and 31 had a good level of knowledge (Table 2).

**Table 2:** Predictor variables and odds ratio (OR) both in binary logistic regression and multivariate analysis.

Variable	Level of knowledge		OR (95% CI)	
	Good knowledge	Poor knowledge	COR (95% CI)	AOR (95% CI)
Sex				
Male	31 (27.4%)	82 (72.6%)	1	
Female	56 (40.6%)	138 (59.4%)	0.932 (0.556, 1.562)	
Age				
18-24	22 (28.9%)	54 (71.1%)	0.433 (0.115, 1.628)	
25-54	53 (29.8%)	125 (70.2%)	0.416 (0.117, 1.480)	
55-64	9 (27.3%)	24 (72.7%)	0.471 (0.111, 2.000)	
65 and above	3 (15%)	17 (85%)	1	
Educational status				
None (illiterate)	19 (17.3%)	91 (82.7)	3.381 (1.390, 8.225)	1.065 (0.224, 5.055)
Can read and write	12 (36.4)	21 (63.6%)	1.235 (0.444, 3.440)	0.434 (0.89, 2.115)
Elementary school	71 (17.9%)	32 (82.1%)	3.227 (1.072, 9.716)	1.569 (0.315, 7.812)
High school	19 (36.5%)	33 (63.5)	1.226 (0.484, 3.107)	0.466 (0.113, 1.925)
College graduate	18 ((40.9%)	26 (59.1)	1.020 (0.393, 2.643)	0.703 (0.238, 2.079
University graduate	12 (41.4%)	17 (58.6%)	1	1
Residence				
Urban	55 (33.5%)	109 (66.5)	1	1
Rural	32 (22.4%)	111 (77.6%)	0.57 (0.343, 0951)	0.625 (0.239, 1.633)
Occupation				
Government employee	28 (46.7%)	32 (53.3%)	1	1
Private business	22 (35.5%)	40 (65.5)	0.291 (0.134, 0.632)	1.829 (0.545, 6.141)
Farmer	23 (19.8%)	93 (80.2)	0.463 (0.211, 1.014)	5.788 (1.235, 27.128)
Other	14 (20.3%)	55 (79.7%)	1.029 (0.489, 2.164)	6.409 (1.650, 24.893)
Previous anesthesia history				
Exposed (Yes)	58 (43.3%)	76 (56.7%)	1	1
Non exposed (No)	29 (16.8%)	144 (83.2)	0.264 (0.156, 0.446)	10.263 (2.236,47.094)**
Frequency of exposure				
Once	40 (40.8%)	58 (59.2%)	1.740 (0.497, 6.093)	2.402 (0.614, 9.396)
Twice	12 (48%)	13 (52%)	1.300 (0.313, 5.393)	1.737 (0.371, 8.125)
Three or more time	6 (54.5%)	5 (45.5%)	1	1

** = significant.

### 5.4 Perception related questions

Our questionnaire contains nine questions to assess patients' perceptions related to the role of anesthetists outside the OR (Table 3). Patients were asked to say yes or no about their perception regarding the role of anesthetists in the recovery or wards when they face breathing difficulties and 172 (56%) patients perceived that the anesthetist has a role, 129 (42%) said no and the rest 6 (2%) said they don't know. Out of the 172 respondents who said yes, about 86 (28%) felt that the anesthetist can help them by administering oxygen through a face mask, 11 (3.6%) patients perceived the anesthetist can play a role by inserting a tube into their trachea to facilitate their oxygenation, 10 (3.3%) patients think that the anesthetist can do tracheostomy, 2 (0.6%) patients perceived he/she can do all of the mentioned tasks and the rest 63 (20.5%) responded that they don't know anything about the tasks done by the anesthetist. Regarding the role of anesthetists in the ER 165 (53.7%) patients perceived that the anesthetist can have a role in treating patients admitted to the ER. And in the ICU patients agreed that anesthetists have a role (164, 53.4%) and out of these respondents 29 (9.4%) said supporting the breathing and ventilation of critically ill patients might be the possible task that can be done by the anesthetist, 78 (25.4%) perceived that administering a sedative, anesthetic and analgesic medications might be the anesthetists' role, and the rest 27 (8.8%) respondents felt that anesthetists can play a role in resuscitation of patients in the ICU like doing cardiopulmonary resustation. Related to the role of anesthetists in treating non-surgical pain about 188 (61.2%) respondents felt that yes there is something that can be done by the anesthetist. Table 3 summarizes all the responses that are related to patients' perceptions about the role of anesthetists outside the operation theatre (Table 3).

**Table 3:** Participants' perception responses.

Questions	Frequency	Percent
If you face breathing difficulty either in the recovery or in the wards, do you think the anesthetist has a role to treat your problem?		
Yes	172	56%
No	129	42%
I don't know	6	2%
What are the possible tasks that can be performed by the anesthetist in the recovery/ward?		
Administering oxygen through the face mask	86	28%
Inserting a tube into your trachea to facilitate your ventilation	11	3.6%
Doing tracheostomy	10	3.3%
Has all the above roles	2	0.6%
I don't know	63	20.5%
Do you think anesthetists have a role in the ER?		
Yes	165	53.7%
No	32	10.4%
I don't know	110	35.8
Do you think the anesthetists have a role in the care of critically ill patients in the ICU?		
Yes	164	53.4%
No	129	42%
I don't know	14	4.6%
What is your opinion regarding the tasks that the anesthetist can do in the ICU?		
Supporting the breathing and ventilation of patients	29	9.4%
Administering sedative, anesthetic and anti pain medications	78	25.4%
Has a role in resuscitation of patients	27	8.8%
Do anesthetists have role in managing non-surgical pain?		
Yes	188	61.2%
No	37	12.1%
I don't know	81	26.4%
Who do you think is the chief of the OR?		
The surgeon	201	65.5%
The anesthetist	34	11.1%
The nurse	18	5.9%
I don't know	54	17.6%
Description of the relationship between the surgeon and the anesthetist		
The anesthetist performs his job under the surgeon's order	72	23.5%
The surgeon performs his job under the anesthetist's order	42	13.7%
Each has different roles	125	40.7%
I don't know	68	22.1%
What do you feel about your safety when you are put under anesthesia?		
I feel safe	93	30.3%
I don't feel any safety	82	26.7%
I'm not so sure (I don't know) what will happen	102	33.2%
I don't have any safety issues	30	9.8%

## 6 Discussion

This study was conducted to assess the patients' level of knowledge on anesthesia and the role played by anesthetists in the perioperative care of patients. Accordingly, we have found that patients had limited knowledge about the field of anesthesiology and the vital role played by anesthesia professionals in patient care both in and outside the operation theatres. Variables such as occupation and previous anesthesia history were found to be significantly affecting the level of patients' knowledge.

Previous anesthesia exposure and level of knowledge are significantly associated in our study and this finding is in line with a survey study done in Turkey where the authors have found that patients who had an anesthesia history have more information about anesthesia. Depending on their findings, they have suggested that providing adequate information about anesthesia could reduce pre-operative anxiety thereby improving surgical outcomes [18]. The findings of our study may be explained by the fact that our institution is a teaching hospital in which the department of anesthesia is always giving great attention to pre-anesthetic assessment to be part of their education both for undergraduate and post-graduate anesthesia students. These students along with their senior instructors spend adequate time in informing, preparing, and optimizing surgical patients. By doing so, patients may get all the details about their anesthetists, anesthesia techniques (options), medications, and the possible side effects and complications. But our study is in contrast with the finding of a cross-sectional study done by Robert D. In this study they did not find any association between previous surgery/anesthetic encounters and knowledge of the roles of anesthetists. Accordingly, the authors have recommended that anesthetists should therefore inform and educate patients about their specialty and their roles, not only during preoperative but also postoperative visits (6).

We have found a statistically significant association between the occupation of participants and their level of knowledge. This finding may not be supported by evidence as there are no data that claim a significant association between patients' occupational status and their level of anesthesia knowledge. Possibly, this different finding in the present study can be explained by the logic that most government officials in Ethiopia in general and in our setup, in particular, have easy access to information about the health sector as these persons may get the chance to participate in different health-related meetings and conferences. Most government institutions in Ethiopia have broadband internet access that these officials can extract information related to anesthesia easily.

It's surprising that in the present study there was no statistically significant association between the level of knowledge and educational status of participants. This is in contrast with most research findings around the world but a cross-sectional study done in Nigeria supports our finding in which education level had no impact on their level of knowledge about anesthesia and anesthetists [11].

Another self-administered structured questionnaire evaluation by Seetharaman H. et al. Strengthens this idea. They have claimed that although many countries in the Caribbean have a good literacy rate, many patients do not want to know details about anesthesia care and the role of their anesthetists [19]. Additionally another prospective questionnaire based study done in Nepal adds an important point to further the idea that educational status doesn't affect anesthesia knowledge [10]. The possible explanation for our finding might be that anesthesiology is a new specialty in our country that whenever someone wants to know about medical services, specialties like internal medicine, surgery, gynecology, and pediatrics come at the front of everyone's arm amentarium. When patients require surgical interventions they only know that the surgeon can do everything for their care and anesthetists and anesthesia services are still hidden. So even though the educational status of the patients is high the possibility that they search for detailed information about anesthesia is limited. Additionally, as an information source majority of the people including the educated ones depends on Social media, television, and radio in which the focus of these information sources is politics, sport news, and other issues. Currently, many government and private media in Ethiopia are inviting different health professionals including internists, surgeons, oncologists, and even physiotherapists but the possibility of anesthetists being invited by these media is rare. Due to this reason, anesthetists might not get the window to elaborate on the details of the profession and the vital role played by anesthetists in the perioperative care of surgical patients to the public.

Regarding percipation of patients about 56% patients felt that the anesthetist could have a role in the management of patients who experienced breathing difficulties outside of the operation room. This result is in contrast with the findings of research done in Turkey where only 10% of patients responded that anesthetists control the patients in the post-operative care unit (18, 20). It may be encouraging to get such credit from patients and this could be due to the reason that patients in our post-anesthesia care unit, which we call the recovery room, may have observed anesthetists being consulted and involved in airway management and related tasks.

We have also interviewed our patients for their response regarding the importance of anesthetists in the emergency room (ER) and our patients had limited awareness about the role of anesthetists in the ER compared to the findings of prospective study done by K. Ferreira and colleagues where about 70.5% of their patients knew that the anesthetist can work in the ER. This discrepancy can be due to the reason that the ER in our hospital is not well organized and anesthetists are not assigned regularly except for some rare consultations. The other possible reason might be we have interviewed patients admitted to the surgical, orthopedic, and gynecology wards sparing the patients in the ER (21).

Respondents awareness about role of anesthetists in the ICU were found to be limited and it has been found, in a study, that about 72.5% of patients answered that anesthetists can work in the ICU. Even though our department has a regular duty schedule in the ICU patients are still not aware that we have a role. Again these could have been a result of excluding ICU patients from our sample [20,21].

The final perception-related question was about how safe do they feel to be put under anesthesia. Accordingly, only 1/3 of patients were not worried about safety. A cross-sectional survey study done in the Caribbean countries revealed similar results with our findings where about 28.3% of patients reported they feel safe. This might be resulted from a lack of delivering every detail on what will happen to patients starting from the time that patients are told about anesthesia, when they are under anesthesia, and after the anesthesia is completed. This would alleviate patients' unnecessary fears and anxiety contributing to a better surgical outcome [2].

## 7 Limitations

As a limitation, this study is institution-based which could limit its generalizability and the cross-sectional nature of the study would also limit its ability to establish a temporal relationship.

## 8 Conclusion and recommendations

As a conclusion, we have found patients have very limited knowledge and perception regarding anesthesia and the role of anesthetists. The advantage of achieving good patient and public recognition of anesthesia and the role of an anesthetist is threefold. First, we can have patients who are well informed of their care which helps them to be free of any unnecessary fear and anxiety and this improves the surgical outcome helps patients to give value for the anesthetic services they got. The second advantage is this wide public recognition and acceptance will boost the satisfaction of anesthetists. Finally, this recognition will attract young students to be anesthetists as Ethiopia is one of the countries with a severe shortage of anesthetists in Africa.

Doing a very extensive pre-anesthetic evaluation of patients could be one way of achieving good recognition by patients. We need to broadcast and elaborate about the anesthesiology and the important role played by anesthetists in the care of patients in a hospital.

## Availability of data and materials

The data generated and analyzed will be available on a reasonable request from the corresponding author.

## Provenance and peer review

Not commissioned, externally peer-reviewed.

## Ethical approval

This study was approved by the Ethics Committee of University of Gondar Comprehensive Specialized Hospital and was performed in accordance with the Helsinki Declaration of 1964 and later amendments. Informed written consent was obtained from each study subject after clear explanation about the objectives and purposes of the study. Participants were informed of their right to refuse to participate in the study at any time. Confidentiality was ensured by avoiding personal identification on questionnaires and by keeping the questionnaires locked.

## Sources of funding

No funding was received.

## Author contribution

This work was carried out in collaboration among all authors. NRA, ABG participated in data entry, analysis, interpretation, manuscript preparation and providing the final version of the study. NSE, YAN participated in conception of the design, proposal writing and edition, contributed in interpretation of the results and revised the paper at the final version. TBM contributed in edition of the final version, contributed in analysis and interpretation. All authors read and approved the submitted manuscript.

## Registration of research studies

1.Name of the registry: Research registry
2.Unique Identifying number or registration ID: 7699
3.Hyperlink to your specific registration (must be publicly accessible and will be checked): https://www.researchregistry.com/browse-the-registry#home/

## Guarantor

Nurhusen Riskey Arefayne (NRA), Amare Belete Getahun (ABG), Tadesse Belayneh Melkie (TBM), Nigussie Simeneh Endalew (NSE), Yonas Adissu Nigatu (YAN).

## Consent

Participants were well informed and agreed with no benefit obtained.

## Author contribution

This work was carried out in collaboration among all authors. NRA, ABG participated in data entry, analysis, interpretation, manuscript preparation and providing the final version of the study. NSE, YAN participated in conception of the design, proposal writing and edition, contributed in interpretation of the results and revised the paper at the final version. TBM contributed in edition of the final version, contributed in analysis and interpretation. All authors read and approved the submitted manuscript.

## Declaration of competing interest

There is no conflict of interest.
